# A54 SHP2 FUNCTION IN NORMAL AND *APC*-MUTANT INTESTINAL EPITHELIAL CELLS

**DOI:** 10.1093/jcag/gwad061.054

**Published:** 2024-02-14

**Authors:** A Côté-Biron, J Gagné-Sansfaçon, N Rivard

**Affiliations:** Universite de Sherbrooke, Sherbrooke, QC, Canada; Universite de Sherbrooke, Sherbrooke, QC, Canada; Universite de Sherbrooke, Sherbrooke, QC, Canada

## Abstract

**Background:**

Somatic *SHP2* gain-of-function mutations were found in some colorectal tumours. However, its function in colorectal epithelium remains to be determined. SHP2 is a tyrosine phosphatase necessary for RAS/MAPK pathway activation by most, if not all, tyrosine kinase receptors, as well as by GPCRs and cytokine receptors. SHP2 can also regulate the PI3K, Jak/Stat, RhoA and Hippo pathways in different cell settings. As a result, SHP2 can control cellular processes including growth, migration and differentiation. Previously, we observed that SHP2 levels are enhanced in human colorectal tumours, notably in late adenomas. Likewise, SHP2 silencing inhibited ERK activation and tumour properties of established human CRC cell lines. Hence, these findings support a model in which SHP2 activation promotes tumorigenesis in the intestinal epithelium. But how?

**Aims:**

To analyze the exact cellular and molecular mechanisms underlying SHP2 pro-oncogenic function in intestinal epithelial cells.

**Methods:**

The impact of SHP2 pharmaco-inhibition (RMC-4550 and/or SHP099) on intracellular signaling, cell cycle, proliferation and survival was evaluated in three complementary models: 1) HIEC-6 cells, normal human fetal intestinal crypt-like cells; 2) organoids derived from normal mouse small intestine or *Apc*^*Min*/+^ tumors; 3) Caco-2/15 cells, colorectal cancer cells with *APC* mutation.

**Results:**

1- Pharmaco-inhibition of SHP2 blocks proliferation of HIEC-6 cells (loss of pRb phosphorylation, decreased EdU staining) and induces cell enlargement and darkening of the outside of perinuclear area. This phenotype was reminiscent of cellular senescence, and we indeed observed increased senescence-associated β-galactosidase activity (SA-β-Gal) and *INK4A* gene expression. **2-** As expected, SHP2 inhibition in normal mouse organoids resulted in a reduction in the number of protrusions and EdU positive cells. RNA sequencing was performed, and gene set enriched revealed significant down regulation of several Ras signaling-dependant genes including cell cycle regulated genes (E2F targets, G2/M checkpoint) and Myc targets 48h after SHP2 inhibition when compared to DMSO treated organoids. Genes associated with PI3K signaling were also diminished in SHP099 treated organoids but no significant modulation of genes associated with Wnt/b-catenin signaling was observed. **3-** Notably, SHP2 pharmaco-inhibition in Caco-2/15 cells as well as *APC-*mutant organoids rapidly induced cell death associated with increased expression of stem cell markers.

**Conclusions:**

These results suggest that SHP2 is required for proliferation of normal IEC and protects against senescence. Notably, its inhibition restrains the proliferative and tumorigenic potential conferred by *APC* inactivation. Overall, our data suggest that SHP2 could represent a potential target to counter colorectal tumour initiation.

**Funding Agencies:**

CIHRFRQS

